# Lack of germline A339V mutation in thyroid transcription factor-1 (TITF-1/NKX2.1) gene in familial papillary thyroid cancer

**DOI:** 10.1186/1756-6614-3-4

**Published:** 2010-08-11

**Authors:** Silvia Cantara, Serena Capuano, Caterina Formichi, Milena Pisu, Marco Capezzone, Furio Pacini

**Affiliations:** 1 Section of Endocrinology & Metabolism, Department of Internal Medicine, Endocrinology & Metabolism and Biochemistry, University of Siena, Siena, Italy

## Abstract

Thyroid cancer may have a familial predisposition but a specific germline alteration responsible for the disease has not been discovered yet. We have shown that familial papillary thyroid cancer (FPTC) patients have an imbalance in telomere-telomerase complex with short telomeres and increased telomerase activity. A germline mutation (A339V) in thyroid transcription factor-1 has been described in patients with multinodular goiter and papillary thyroid cancer. In this report, the presence of the A339V mutation and the telomere length has been studied in FPTC patients and unaffected family members. All samples analyzed displayed a pattern typical of the homozygous wild type revealing the absence of the A339V mutation. Shortening of telomeres was confirmed in all patients. We concluded that the A339V mutation in thyroid transcription factor-1 (TITF-1/NKX2.1) is not correlated with the familial form of PTC, even when the tumor was in the context of multinodular goiter.

## Findings

Familial non-medullary thyroid cancer (FNMTC), most frequently of papillary hystotype (FPTC), recurs in two or more members of the same family in about 10% of the patients. The clinical form of PTC may be part of a clinical syndrome such as the Adenomatous polyposis of colon (FAP) [1], Cowden syndrome [2], Gardner syndrome [3], Werner syndrome [4] or Carney complex [5] or may be the only disease manifestation. In this case, the large majority of FPTC, no candidate gene(s) has been discovered [6]. Recently we provided evidence that FPTC display the feature of "genetic anticipation" (defined as earlier age at onset of the disease and/or increased severity in successive generations), and, at molecular level, are characterized by the presence of germinal alterations in the telomere-telomerase complex [7,8]. Our hypothesis is that the imbalance in telomerase complex may predispose to acquire a thyroid specific mutation able to trigger thyroid tumorigenesis. Recently, Ngan et al. demonstrated the presence of a new germline mutation (A339V) in thyroid transcription factor-1 (TITF-1/NKX2.1) in patients with multinodular goiter and papillary thyroid cancer [9]. The authors identified the mutation in four out of 20 MNG/PTC patients which developed more advanced tumors compared to MNG/PTC or PTC patients without the mutation. In addition, the mutation was dominantly inherited in two families with members bearing MNG/PTC [9].

**Figure 1:**
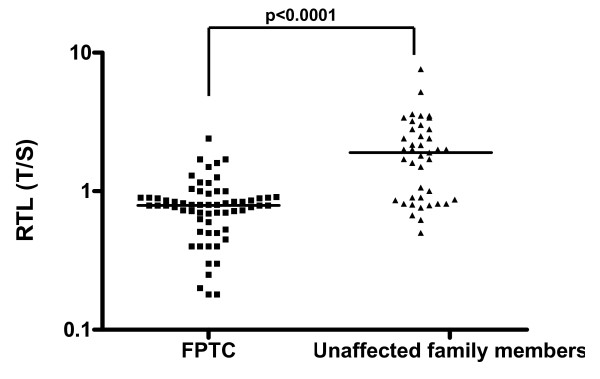
**RTL expressed as T/S of 63 FPTC patients compared to 41 unaffected siblings Statistic by Student's t-test (A)**.

**Figure 2:**
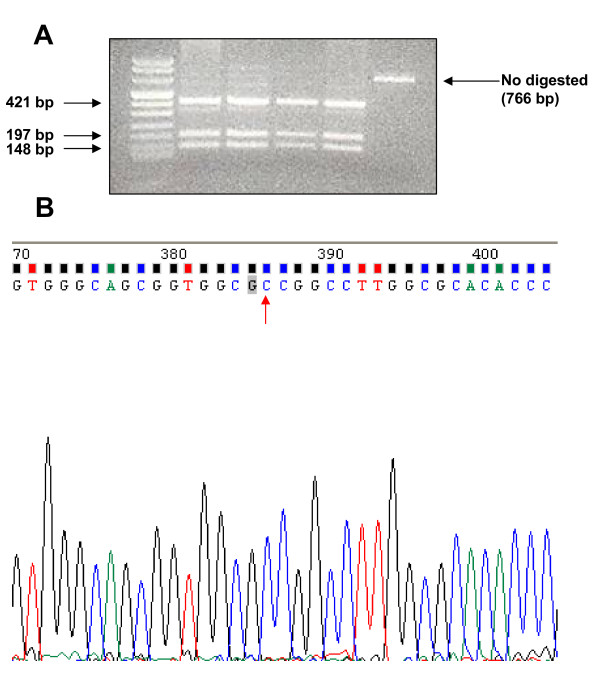
Representative gel illustrating the homozygous wild type digestion pattern showed by FPTC patient and unaffected siblings (A); Representative electropherogram of an FPTC patient showing the absence of mutation(s) in TITF/NKX2.1 gene (B).

In light of these evidences, we investigated the presence of A339V mutation in patients with the occurrence of FPTC and short telomeres. Out of 408 patients affected by differentiated thyroid carcinoma (DTC), followed in the Section of Endocrinology of University of Siena (Italy) from 1978 to 2009 (306 females and 102 males; ratio F/M: 3/1, ranging 8-84 yrs), 63 patients (15.4%), belonging to 38 kindred, showed the familial recurrence of the disease possibly configuring the features of familial thyroid cancer (FTC). All patients with FTC had the papillary hystotype (FPTC), 15 of them (23.8%) presented the follicular variant and 1 (1.6%) the warthin-like variant. Of these patients, 40 (63.4%) had a parent-child relationship (22 kindred), 19 (30.2%) had a sibling relationship (13 kindred) and 4 (6.4%) had an uncle-niece relationship (3 kindred). Age at diagnosis of FPTC group was 46 ± 15.5 yrs (range: 15-78 yrs). Age at diagnosis of probands was 54.3 ± 9.3 (range 25-77 yrs) and that of affected familial members was 42.9 ± 15.7 (range 15-73 yrs). No thyroid cancer associated syndrome was present clinically in our patients. Clinical features of FPTC patients are reported in Table 1. In addition, we analyzed 41 unaffected family members of FPTC patients (14 males and 27 females, mean age: 44.4 ± 19 yrs, range: 14-62 yrs) belonging to 25 kindred. Among FPTC patients, 23 (36.5%) had a single nodule whereas 40 (63.5%) patients had a history of multinodular goiter. At the time of the study 45 (71.4%) were free of disease, 12 (19.1%) showed persistent disease and 6 (9.5%) were operated less than 1 year ago (follow-up not available). In the group of 41 unaffected family members, 33 (80.6%) patients had no evidence of thyroid nodule, 4 (9.7%) showed a multinodular goiter and 4 (9.7%) had a single nodule. In all cases the FNAB indicated the presence of benign lesions. We measured telomere length by Q-PCR [10] assay on genomic DNA of all subjects and compared the differences in telomere length by Student's t-test. As shown in figure 1, RTL was significantly (p < 0.0001) shorter in FPTC patients (mean ± SD: 0.8 ± 0.04, range: 0.18-2.4) compared to unaffected siblings (mean ± SD: 2.04 ± 0.2, range: 0.7-7.6). To identify whether TITF/NKX2.1 germline point mutation (A339V) was involved with FPTC development, genomic DNA belonging to FPTC patients and unaffected siblings, was amplified using the GC-Rich PCR system followed by digestion of PCR products by restriction enzyme NaeI. This enzyme cut in correspondence of 5'-GCCGGC-3' sequence thus leading to the formation of these patterns: A) homozygous wild type characterized by three fragments of 421, 197 and 148 bp, respectively; B) mutated homozygous characterized by two PCR products of 618 and 148 bp, respectively and C) heterozygous characterized by four fragments at 618, 421, 197 and 148 bp, respectively. As shown in figure 2A, after digestion with the enzyme all the analyzed samples displayed a pattern typical of homozygous wild type. To confirm this result, samples were amplified twice and PCR products directly sequenced. As reported in figure 2B no mutation was found in all samples.

**Table 1:** Clinical features of FPTC patients

	Patients (n = 63)
**Age at diagnosis (yr)**	
Mean ± SD	46 ± 15.5
Range	15-78

**Sex**	
No. of females (%)	47 (74.6)
No. of males (%)	16 (25.4)

**Histotype**	
No. of papillary (%)	47 (74.6)
No. of papillary follicular variant (%)	15 (23.8)
No. of papillary warthin-like (%)	1 (1.6)

**TNM**	
No. of T1-T3 N0 M0(%)	40 (63.5)
No. of T1-T3 N1 M0 (%)	13 (20.6)
No. of T1-T3 N0-N1 M1 (%)	1 (1.6)
No. of T4 N0-N1 M0 (%)	3 (4.7)
Not available	6 (9.6)

**Outcome**	
Remission (%)	45 (71.4)
Persistent disease (%)	12 (19.1)
Not evaluated (%)	6 (9.5)

In conclusions, in this study we analyzed 63 FPTC patients for the presence of the A339V mutation in thyroid transcription factor-1 (TITF-1/NKX2.1) in order to correlate that mutation with the predisposition to develop familial form of thyroid cancer demonstrated by shorter telomere length which may contribute to genetic instability and thyroid cancer development. Our results confirmed that FPTC patients have significantly short telomeres compared to unaffected family members. The reason of the predisposition to develop thyroid cancer in patients displaying the imbalance in telomerase complex has not been identified yet. The A339V mutation in thyroid transcription factor-1 (TITF-1/NKX2.1) might be a candidate gene in view of recent findings [9]. All FPTC patients displayed a genetic profile typical of homozygous wild type demonstrating that A339V mutation is not necessarily associated with the development of familial form of thyroid cancer even when the tumor was in the context of multinodular goiter.

## Competing interests

The authors declare that they have no competing interests.

## Authors' contributions

SC carried out the molecular genetic studies and wrote the manuscript, SC contributed to perform the experiments, CF helped to collect clinical patient's data, MP helped with DNA extraction, MC revised the clinical data and FP revised the manuscript and coordinated the experiments.

All authors read and approved the final manuscript.

## References

[B1] Giardiello FM Offerhaus GJA Lee DH Krush AJ Tersmette AC Booker SV Kelley NC Hamilton SR Increased risk of thyroid and pancreatic carcinoma in familial adenomatous polyposis Gut 1993 34 1394 1396 10.1136/gut.34.10.1394 8244108 PMC1374548

[B2] Liaw D Marsh DJ Li J Dahia PLM Wang SI Zheng Z Bose S Call KM Tsou HC Peacocke M Eng C Parsons R Germline mutations of the PTEN gene in Cowden disease, an inherited breast and thyroid cancer syndrome Nat Genet 1997 16 64 67 10.1038/ng0597-64 9140396

[B3] Kelly MD Hugh TB Field AS Fitzsimons R Carcinoma of the thyroid gland and Gardner's syndrome Aust N Z J Surg 1993 63 505 9 10.1111/j.1445-2197.1993.tb00439.x 8498926

[B4] Goto M Miller RW Ishikawa Y Sugano H Excess of rare cancers in Werner syndrome (adult progeria) Cancer Epidemiol Biomarkers Prev 1996 5 239 246 8722214

[B5] Stratakis CA Courcoutsakies NA Abati A Filie A Doppman JL Carney A Shawker T Thyroid gland abnormalities in patients with the syndrome of spottyskin pigmentation, myxomas, endocrine overactivity, and schwannomas (Carney complex) J Clin Endocrinol Metab 1997 82 2037 2043 10.1210/jc.82.7.2037 9215269

[B6] Sturgeon C Clark OH Familial nonmedullary thyroid cancer Thyroid 2005 15 588 93 10.1089/thy.2005.15.588 16029126

[B7] Capezzone M Marchisotta S Cantara S Busonero G Brilli L Pazaitou-Panayiotou K Carli AF Caruso G Toti P Capitani S Pammolli A Pacini F Familial non-medullary thyroid carcinoma displays the features of clinical anticipation suggestive of a distinct biological entity Endocr Relat Cancer 2008 15 1075 81 10.1677/ERC-08-0080 18832444

[B8] Capezzone M Cantara S Marchisotta S Filetti S De Santi MM Rossi B Ronga G Durante C Pacini F Short telomeres, telomerase reverse transcriptase gene amplification, and increased telomerase activity in the blood of familial papillary thyroid cancer patients J Clin Endocrinol Metab 2008 93 3950 7 10.1210/jc.2008-0372 18664542

[B9] Ngan ESW Lang BHH Liu T Shum CKY So MT Lau DKC Leon TYY Cherny SS Tsai SY Lo CY Khoo US Tam PKH Garcia-Barcelo MM A Germline mutation (A339V) in thyroid transcription factor-1 (TITF-1/NKX2.1) in patients with multinodular goiter and papillary thyroid carcinoma JNCI 162-75 101 10.1093/jnci/djn471 19176457

[B10] Cawthon RM Telomere measurement by quantitative PCR Nucleic Acids Research 2002 30 47 10.1093/nar/30.10.e47 12000852 PMC115301

